# How enterprise interactions in innovation networks affect technological innovation performance: The role of technological innovation capacity and absorptive capacity

**DOI:** 10.1371/journal.pone.0282540

**Published:** 2023-03-02

**Authors:** Mengjuan Fan, Wu Huang, Shengxu Xiong

**Affiliations:** 1 School of Business Administration, Zhongnan University of Economics and Law, Wuhan, Hubei, China; 2 Department of Logistics Management and Engineering, Nanning Normal University, Nanning, Guangxi, China; University of Hertfordshire, UNITED KINGDOM

## Abstract

Current research on the impact of innovation networks focuses on the web and inter-organizational layers, with less consideration of individual behavior at the firm level. Interaction is an active action strategy that firms take when dealing with the external environment. Therefore, this study explores the mechanism of enterprise interaction on innovation development from the perspective of an innovation network. And measures enterprise interaction in three dimensions: affective interaction, resource interaction, and management interaction. The empirical results indicate that the three dimensions of enterprise interaction contribute significantly to technological innovation performance, and the realization of this role requires technological innovation capabilities (technological research and development capabilities, technological commercialization capabilities) to play a partially mediating role. The moderating effect of absorptive capacity between resource interaction, management interaction, and technological innovation capability is significant; however, the moderating effect between affective interaction and technological innovation capability is statistically insignificant. This study promotes the development of interaction theory to a certain extent, which helps enterprises build appropriate industrial chains in innovation networks and achieve rapid development.

## Introduction

Economic globalization has promoted the division of labor and collaboration in global value chains, and the increasing trend of the multi-polarization of innovation has accelerated the formation of globalized innovation networks [1]. Open, cooperative, and networked innovation models replace traditional closed, independent, and linear innovation methods and processes [2, 3]. Meanwhile, in the fierce business competition, technological innovation has become a critical factor that restricts the survival and development of enterprises. To seize the new historical opportunities, enterprises need to integrate into the innovation network with a more open vision and proactive attitude [4]. Through active interaction with network members, they can fully absorb and integrate external resources and improve the quality and efficiency of enterprise technological innovation in open cooperation [1, 5].

Research on the impact of innovation networks on firms’ innovation performance can be broadly divided into two categories. One category is from a social network science perspective, focusing on the impact of the looseness of network structure [6], the centrality of network location [7, 8], structural holes [9], and the strength of network linkages [10] on firms’ innovation activities. The other category is from the perspective of relational research, exploring the role of various relationship types, such as formal and informal relationships [11], horizontal and vertical relationships [12, 13], multi-stage and multi-level cooperation [14, 15], and cooperation with different goals [5, 16], on the innovation performance of enterprises. The above studies mainly focus on the network and inter-organizational levels but consider less the individual behaviors of firms in innovation networks. The impact of firms’ interactive behaviors on innovation development has also been ignored. Therefore, it is necessary to investigate further the role of enterprise interaction behavior in innovation networks on innovation activities [8].

In addition, the influence of inter- enterprise interaction on firm performance has received much attention from the academic circle. Interaction is essential in establishing, maintaining, and utilizing external relationships. Existing studies mainly focus on two aspects: First, in buyer-supplier relationships, an excellent interactive relationship is more conducive to smooth communication and the realization of bilateral transactions [17] and improves the cooperation performance of enterprises [18]. Even in the asymmetric relationship between small suppliers and large buyers, small suppliers have more opportunities to gain positioning advantages and realize value improvement through interaction [19, 20]. Second, in the service-oriented customer relationship, frequent interaction will improve customer satisfaction [21] and facilitate enterprises to develop new products and services [22] more targeted to improve customer performance [23].

To sum up, the role of interaction has been well demonstrated in binary relationships such as firm-supplier and firm-customer. However, the existing literature is primarily qualitative research due to the lack of mature measurement scales [24, 25]. The research results mainly focus on the bilateral relationship, cooperation performance, customer performance, and other aspects [20, 26, 27]. In addition, there are few pieces of literature on the impact of interaction on actors at the micro level [25, 28]. In particular, empirical studies on how interaction affects the technological innovation performance of focus firms are rare, and there is still a lot of research space.

Based on these, this study asks three questions: 1) How do firms in innovation networks interact? (2) How do inter- enterprise interactions affect the technological innovation performance? 3) Is the impact of interactions on innovation still influenced by their factors? Existing studies do not answer these questions, and there need to be more empirical studies on the relevant mechanisms of action [25].

To answer these questions, this study presents an empirical study of the interaction behavior of focal firms in the context of an innovation network perspective. Interaction in innovation networks is a way for firms to gauge their resources fully and to activate and exploit external resource links [29, 30]. Based on this theoretical perspective and following the theoretical framework of the resource strategy view, "resources determine capabilities, and capabilities determine performance." This study proposes that enterprise interaction in innovation networks not only has a direct impact on technological innovation performance, but also has an indirect impact on it by enhancing the technological innovation capability of firms. Through theoretical analysis and empirical study of the above perspectives, this paper reveals the intrinsic mechanism by which interactions among firms in innovation networks affect firms’ technological innovation performance.

The innovations of this paper are 1) considering the interaction behavior of firms in innovation networks and refining the connotation and dimensions of enterprise interaction; 2) developing a measurement scale for enterprise interaction to provide measurement tools for subsequent empirical studies; 3) focusing on the participants of interaction and studying the impact of interaction on the technological innovation interaction of focal firms in innovation network scenarios, which to some extent fills the gap of existing studies.

This study will help discover the research value of enterprise interactions in a more open environment and deepen the interaction theory. In addition, it provides a new management perspective for enterprises’ technology innovation practice. It is also significant to guide enterprises to build good network relationships.

## Theoretical background and research hypothesis

### Enterprise interaction in the innovation network

#### Enterprise interaction

In industrial Marketing and Purchasing (IMP), Sheth (1976) provided a comprehensive concept of the buyer-seller interaction process and emphasized that interaction is a two-way communication method [31]. With the deepening of research, people no longer regard enterprise interaction as simply communication or negotiation [32]. It is the process of contacting each other for commercial reasons, obtaining resources needed for development, and influencing each other in contact [23]. The view of resource dependence theory is that when a company’s internal resources are challenging to meet the needs of survival and development, the company has to trade with the external environment. The process of obtaining external resources through communication, negotiation, and negotiation is interaction [33]. Focus enterprises, through interaction, coordination, and external relations, can get various resources needed for enterprise development [29]. Therefore, this paper regards enterprise interaction as a crucial external innovation resource.

#### Enterprise interaction in the innovation network

An innovation network is a relationship network built by enterprises based on the sharing and acquiring of innovation resources [11]. It is also an interactive system formed to facilitate the exchange of information and resources between enterprises [14]. The relationship between enterprises in the innovation network can be horizontal or vertical, formal or informal. In addition, cooperation goals in an innovation network are diverse for specific innovation projects or a simple exchange of resources and information [5, 16]. The above characteristics of an innovation network give members ample space for interaction [34]. Therefore, this study believes that enterprise interaction in an innovation network is a process of acquiring knowledge, utilizing resources, and maintaining network relationships.

### The dimensions of enterprise interaction in innovation network

**Fig 1:**
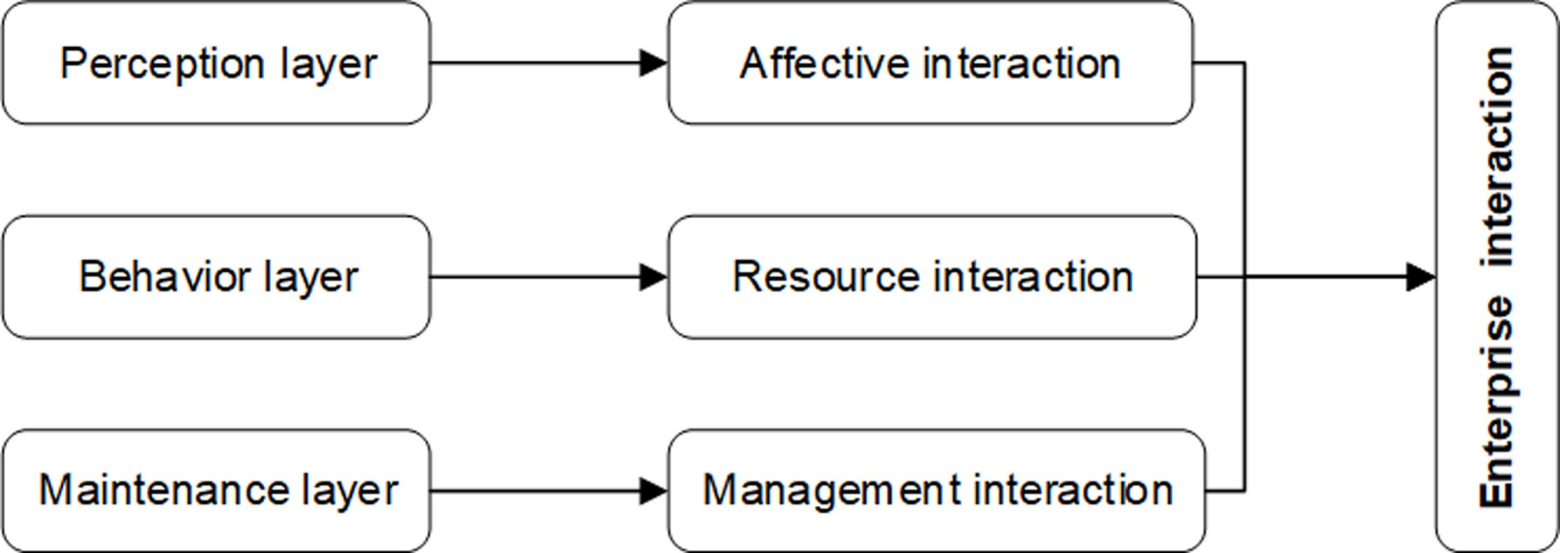
Dimensional analysis of enterprise interaction.

ARA model is usually used in IMP research to understand the process of interaction [32]: The first dimension comprises the ***actors’-bonds*** established between companies in terms of trust vs. distrust, closeness vs. distance, cooperation vs. competition, power and conflict. The second dimension includes the ***activity-links*** that resources and information shared by firms such as goods and services, manufacturing facilities, capital, technology, knowledge, and people. The third dimension comprises the ***resource-ties*** that cooperative activities such as storage, logistics, technology development, sales and marketing activities, and procurement. Further, the ARA model can be derived into three dimensions: affective cognition, specific behavior, and relationship management. The first is the perception level, which is a way to understand the interaction [24, 35, 36], because the interactive behavior of the participants will be affected by the perception of trust and commitment, and the perceptual results will further promote the interaction [19]. The second is the behavior layer. The purpose of business interaction is not simply to develop friendly relations but to realize the exchange and sharing of crucial resource information through ties. The exchange, combination, and redevelopment of resources between enterprises are the substantive interaction processes [29, 37]. Finally, there is the maintenance layer. That is, enterprises organize interactions from the perspective of maintaining and coordinating business relations to realize the smooth progress of cooperative activities and sustainable development [24]. Therefore, this paper divides enterprise interaction into three dimensions: affective interaction, resource interaction, and management interaction (Fig 1).

**Affective interaction** refers to establishing a good relationship foundation and stimulating the vitality and willingness of interaction. Including: 1) Create a good relationship atmosphere and eliminate the distance between enterprises; 2) Active expression of emotion (trust, commitment, etc.); 3) Partners’ recognition of the enterprise, the cooperation relationship, and the ability of both parties.

**Resource interaction** is a process in which enterprises combine, utilize and jointly develop resources to enhance friendly cooperation. These include 1) The exchange and use of resources such as essential goods or services and production or transportation equipment; 2) Sharing and exchange of technology, knowledge, etc.; 3) Utilization of corporate social resources; 4) Reorganization and secondary development of resources among different enterprises.

**Management interaction** is a series of behaviors for enterprises to maintain, optimize, consolidate, and increase the rent of relationships. Including 1) role specification; 2) conflict coordination; 3) relationship maintenance; 4). joint improvement; 5). promote cooperation, etc.

### Enterprise interaction and technological innovation performance

Affective interaction is an actor’-bond between participants in business activities. Tojeiro-Rivero & Moreno (2019) found that good affective interaction between firms is the basis for building good organizational relationships and successful access to resources across boundaries [38]. The stronger the affective interactivity between firms, the higher the intimacy between innovation agents, which will bring higher resource commitment to both interacting parties [39]. High-quality affective interaction is the key to bridging the perception gap between enterprises, which increases identification with the firm’s capabilities and their relationship. It is believed that the other party will take the relationship role seriously and will not act in a way that infringes on the ties [40, 41]. In addition, frequent affective interactions can effectively mitigate opportunistic behaviors between organizations, which improves the efficiency of knowledge transfer between firms and helps achieve higher innovation performance.

Resource interaction in innovation networks is an important way for firms to access rare resources and critical information. Dyer & Singh (1998) argue that competitive advantage comes not only from within the firm but also from resources in the external environment which may be embedded in partnerships and network relationships [42]. Therefore, the more external relations enterprises interact with, the more network resources they can access and use. Resource interaction promotes the flow of knowledge and information in the network, thus activating the connection of different resources and providing more options for the enterprise’s technological innovation activities [43]. Maintaining good resource interaction is conducive to attracting more intellectual and human capital to participate in the firm’s innovation process, reducing the risk of innovation decisions, and increasing the success rate of innovation [22]. In addition, resource interaction can realize deep binding with network members, which not only strengthens network relations but also provides diversified social resources for focus enterprises [29]. Finally, by recombining and reconfiguring numerous resources, new creative enthusiasm is stimulated, and technological innovation performance is driven to improve.

Management interaction is a meaningful way to maintain stable external relations. Management interaction can effectively resolve conflicts and contradictions between partners and promote the smooth development of innovation collaboration activities [44]. In the innovation network, good management interaction can make the relationship coordination more flexible, help enterprises to be recognized by other members, and obtain more opportunities for collaborative innovation. In addition, management interaction helps to establish generally accepted norms of behavior among network members and to develop mutually consistent patterns of living together. It ensures the partnership’s long-term sustainability and the innovation network’s stable operation [35]. But there is a cost to keeping in touch with many businesses. Management interactions also assist firms in relationship screening and eliminating redundant ties. Enable enterprises to invest limited management energy into the most beneficial relationship to promote the rapid improvement of technological innovation performance [45, 46]. Based on the above theoretical analysis, this paper proposes the following hypotheses:

H1a: Affective interaction has a significant positive effect on technological innovation performance.
H1b: Resource interaction has a significant positive impact on technological innovation performance.
H1c: Management interaction has a significant positive impact on technological innovation performance.

### Enterprise interaction and technological innovation capabilities

Technological innovation capability is a firm’s ability to deal with the technological innovation’s mechanism and relationship issues from input to output. It has two dimensions: (1) technology research and development capability (R&D) and (2) technology commercialization capability (TC) [47]. R&D capability refers to a firm’s ability to research, design, and develop new products and process technologies [48, 49], whereas TC capability refers to a firm’s ability to bring new products and processes to the market to realize their commercial value [50, 51]. The enterprise interactions in innovation networks helps to promote R&D capability by obtaining the information, knowledge, technology, and other resources required for innovation. In addition, enterprise interaction also firms broaden the business ecosystem, improve the efficiency and speed of commercializing their own technologies or products, and further enhance the competitive advantage [51]. Frequent and continuous affective interactions between firms in an innovation network enhance mutual trust between firms [19]. First, this mutual trust leads to increased resource commitment and a higher willingness to invest [43], facilitating access to crucial innovation resources and increases innovation investment. Second, affective interactions create an interactive atmosphere that enables access to the knowledge and technical information needed for innovation and increases the possibility of improving technological R&D capabilities [39]. Finally, affective interaction forges a strong communication bond that can lead firms to break away from their own experience and path dependence [52]. Firms will continuously adjust their organizational resources or structure to promote the improvement and development of technological innovation capabilities.

Companies participate in continuous resource interaction in innovation networks, which is conducive to expanding information and resource boundaries as well as providing heterogeneous resources and diversified information for R&D innovation [53]. Second, resource interaction also facilitates corporate innovators’ access to external knowledge, thus stimulating creative inspiration and enhancing innovation capabilities. Furthermore, resource interaction helps enterprises learn how to mobilize and manage the resources of network members [54]. Thus, it creates conditions for them to integrate innovation resources such as R&D, manufacturing, and marketing to enhance their innovation capability [29].

Management interaction is dedicated to the improvement of cooperative relationships. On the one hand, resolving conflicts with other firms will help firms learn solutions to specific problems and improve their ability to coordinate and allocate innovation resources efficiently [53]. On the other hand, maintaining good management interaction with network members helps innovative firms learn from the superior expertise of partner firms. Additionally, it can employ the R&D facilities, production capabilities, and marketing channels of other partner firms to enhance technological research, development, and commercialization innovation capabilities [4, 55]. Based on the above theoretical analysis, this study proposes the following hypotheses:

H2a: Affective interaction has a significant positive impact on R&D capability.
H2b: Resource interaction has a significant positive impact on R&D capability.
H2c: Management interaction has a significant positive impact on R&D capability.
H3a: Affective interaction has a significant positive effect on TC capability.
H3b: Resource interaction has a significant positive impact on TC capability.
H3c: Management interaction has a significant positive effect on TC capability.

### Technological innovation capabilities and technological innovation performance

Recent research based on a capability perspective have found that the performance of organizations varies according to their technological capabilities and that investment in technical capabilities also positively affects their performance [22, 47, 56, 57]. Especially in technology-driven industries, a company’s depends on its ability to introduce a unique product or service to the market [58].

The level of R&D capability dramatically affects the speed, direction, and services of innovation, which determines the innovation output performance of a firm. In addition to having quick access to information, knowledge, technology, and other resources required for R&D activities in a complex environment, firms with strong R&D capabilities also improve their internal knowledge base and gain a competitive advantage in opening new R&D areas [59]. It also has better physical conditions for R&D, more practical technical expertise, and a more helpful team of research developers and innovators [60]. Such companies have a extraordinary ability to integrate multidisciplinary knowledge and create new products and technologies based on the fusion of knowledge [61].

A company’s TC capability is defined as its ability to quickly bring new products or technologies to the market [62]. An enterprise’s more robust TC capability means that it has excellent manufacturing and marketing capabilities, which can turn new products and technologies researched and developed by the enterprise into practical products and processes [50]. In addition, a company with strong TC capability becomes a target for other companies to compete for cooperation in the innovation network. For this enterprise, collaborative innovation will not only reduce R&D costs and risks but also accelerate the speed and efficiency of innovation output and improve technological innovation performance [51]. Therefore, the following hypotheses are proposed:

H4a: R&D capability has a significant positive effect on technological innovation performance.
H4b: TC capability has a significant positive effect on technological innovation performance.

### The mediating role of technological innovation capabilities

According to theories related to resource base, resources are the fundamental cause for the formation of enterprise capabilities and determining performance differences. With the increasing demand for resources and knowledge in enterprise innovation, seeking resources from outside has become the primary choice to alleviate resource constraints. Enterprise interaction is a critical way to obtain valuable external resources [32]. An innovation network has many rich types of resources; enterprises can get diversified innovation resources through interaction. One part can act directly on technological innovation performance, and the other needs to be transformed into capabilities before performing indirectly on innovation performance [33]. Through accumulating knowledge and experience, enterprises can form unique technological innovation capabilities and continue to obtain competitive advantages. From this perspective, enterprise interaction indirectly affects technological innovation performance, and the realization of this effect needs to be mediated by technical innovation ability. In the context of this study, access to innovation-related resources (knowledge, skills, ideas, etc.) through enterprise interaction will promote the improvement of R&D capability and improve the efficiency and speed of TC [63]. Finally, the competitive advantages of enterprises can be further enhanced, and higher technological innovation performance can be created.

In the context of this study, access to innovation-related resources (knowledge, skills, ideas, etc.) through affective, resource, and management interactions will not only contribute to the improvement of technological research and development capabilities but also the efficiency and speed of commercialization of the firm’s technology or products. Finally, the competitive advantages of enterprises can be further enhanced, and higher technological innovation performance can be created. Therefore, the following hypotheses are proposed:

H5a: R&D capability has a mediating role between enterprise interaction and technological innovation performance.
H5b: TC capability has a mediating role between enterprise interaction and technological innovation performance.

### The moderating role of absorptive capacity

Absorptive capacity is an enterprise’s ability to identify, digest, and apply internal and external knowledge resources [64]. It can effectively promote both the absorption of external innovation resources and internal organizational learning and R&D activities, enhancing firms’ R&D capability and the commercialization of new products and technologies [65, 66].

In affective interaction, enterprises with high absorptive capacity can more acutely grasp the effective information generated in the process of interaction and thus carry out innovation activities in a more targeted manner [67]. In resource interaction, enterprises with high absorptive capacity have good screening and digestion of resource information, which will effectively reduce the occupation of repeated and redundant information in the enterprise knowledge base [68]. It helps enterprises overcome capability traps, get rid of path dependency [69], and identify additional avenues for TC. In management interaction, resource integration and knowledge collision will occur frequently. Firms with high absorptive capacity in the process of technology exchange tend to have more robust organizational mechanisms to truly transform information and resources into corporate ownership, which helps accelerate the efficiency of new technology development and the speed of new product commercialization [70, 71]. Based on this, the following hypotheses are proposed:

H6a: Absorptive capacity has a positive moderating effect on the relationship between affective interaction and R&D capabilities.
H6b: Absorptive capacity has a positive moderating effect on the relationship between affective interaction and TC capability.
H6c: Absorptive capacity has a positive moderating effect on the relationship between resource interaction and R&D capability.
H6d: Absorptive capacity has a positive moderating effect on the relationship between resource interaction and TC capability.
H6e: Absorptive capacity has a positive moderating effect on the relationship between management interaction and R&D capability.
H6f: Absorptive capacity has a positive moderating effect on the relationship between management interaction and TC capability.

### Conceptual model

**Fig 2:**
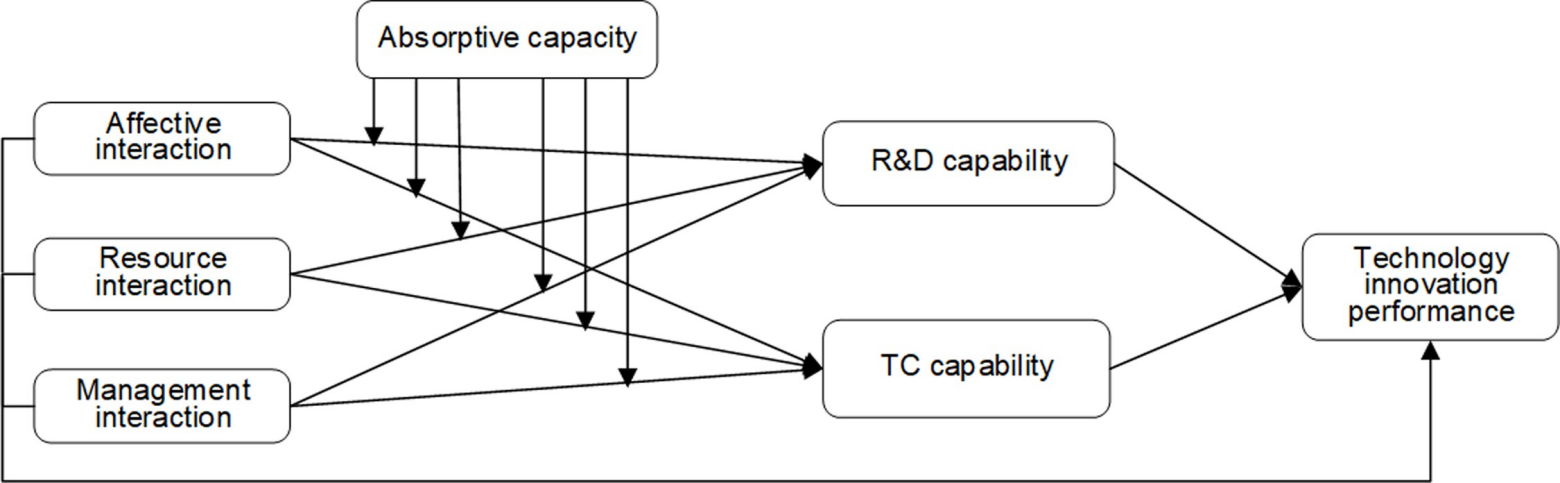
Conceptual model.

This paper studies the influence of enterprise interaction on firms’ technological innovation performance, and analyzes the mediating effect of technological innovation capabilities and the moderating effect of absorptive capacity. Based on the above theoretical analysis and hypotheses, the following conceptual model is proposed (Fig 2).

## Research design

### Samples and data

A questionnaire survey of Chinese firms, including firms from different provinces, of various sizes, industry characteristics, and ownership structures, served as the primary data source for testing the research model and hypotheses. These firms not only have the core technical knowledge of the industry and rich experience in enterprise interaction. In recent years, they have also achieved significant technological innovation performance and are suitable to be the research subjects of this study. Data were collected by field interview, E-mail, and mail. We use field interviews mainly to grasp enterprise members’ understanding of the research topic and delete or modify the items with ambiguous expressions. Follow-up questionnaires were distributed by E-mail and appointment mail.

The questionnaire survey consisted of two steps: the first stage was a pre-study, we sent 200 questionnaires to the target companies and recovered 169 valid ones. Principal component analysis and maximum variance method were used for factor analysis. Items with a factor loading less than 0.5 were deleted, and the remaining items constituted the formal questionnaire of the study. The second stage was the formal research. We have issued a formal questionnaire of 500, recovery of 414 questionnaires, and 358 valid questionnaires; the effective recovery rate was 71.6%. In the valid sample, the surveyed enterprises were mainly distributed over 25 provinces, including Beijing, Shanghai, Guangdong, Zhejiang, etc. 54.19% of enterprises from high-tech industries (such as electronics, telecommunications, pharmaceuticals, new energy, new materials, etc.), and 45.81% are traditional manufacturing industries (such as machinery, metallurgy, building materials, etc.).

To avoid homogeneous bias caused by using the same data source during questionnaire collection. In the design of items, we use concise expressions and anonymous ways to fill in. The prediction variable and the criterion variable are measured separately. In addition, we paid attention to the separation of sampling time and conducted multiple surveys on sample enterprises. The whole collection cycle was as long as five months. Finally, as Podsakoff et al. (2003) suggested controlling for the effects of an unmeasurable latent method factor was used to test the common method bias [72]. We first calculated the original seven-factor model fitting index (χ^2^ = 673.191, p≈0.000, df = 413, χ^2^/df = 1.630, CFI = 0.962, TLI = 0.958, RMSEA = 0.042, RMR = 0.08) and then added a method factor to the seven-factor model to become the eight-factor model (χ^2^ = 584.877, p≈0.000, df = 482, χ^2^/df = 1.213, CFI = 0.971, TLI = 0.964, RMSEA = 0.039, RMR = 0.068). The results show that the model fitting index is not significantly improved (△CFI = 0.009, △TLI = 0.006, △RMSEA = -0.003, △SRMR = -0.012), and all indexes did not change more than 0.05. In addition, CFA results show that the single-factor model is poorly fitted (χ^2^ = 3624.926, χ^2^/df = 8.352, CFI = 0.537, TLI = 0.504; RMSEA = 0.144; SRMR = 0.235). Therefore, it can be concluded that this study’s common method bias is not severe.

### Measure

#### Enterprise interaction

We closely adhered to the scale development method Hinkin (1998) proposed to develop the measurement scale for enterprise interaction [73]. In the first step, the measurement items were generated and revised, primarily from existing literature compilation (27 items) [22, 29, 37, 43, 74–79] and open-ended questionnaire coding (14 items), yielding a total of 41 items. In the second step, following the opinions of three experts, 21 items were retained after adjusting the content and structure of the questionnaire. Then Q-sort was used to re-categorize all question items [80], delete those with less than 80% consistency in evaluation, and complete the division of the initial scale, retaining 18 items. In the third step, the scale reliability was measured with exploratory factor analysis, and deleted items with factor loadings lower than 0.5. We also verify the stability of the factor structure and the convergent validity and discriminant validity of each dimension. Finally, we completed a enterprise interaction scale containing 15 items. (see S1 Appendix in S1 File).

All other variables were appropriately adapted to the needs of the study based on existing scales. ***Technological innovation performance*** was measured using four items, which mainly reflected the firm’s product innovation process innovation performance [81, 82]. ***R&D capability*** was measured using four items that reflected four aspects of a firm’s R&D investment, staffing, innovation mechanism, and flexibility of R&D capability [49, 83]. ***TC capability*** was measured using four items that reflected a firm’s technology breadth, market scope, and speed of commercialization [50, 83]. ***Absorptive capacity*** was measured by using the proposed four-item scale, which mainly responds to acquiring, absorbing, transforming, and utilizing external knowledge [84].

#### Control variables

Because technological innovation performance is also influenced by the size of the company, the years of operation, and average annual sales revenue, these three factors were used as control variables in this study.

## Data analysis and results

### Data analysis

#### Reliability and validity analysis

After the completion of the data collection, exploratory factor analysis was used to confirm factor loadings, and reliability was assessed using Cronbach’s alphas (see S1 Appendix in S1 File). All scales had an alpha of above 0.5, indicating that the scale had good reliability. As the questionnaire was designed based on an in-depth literature review and field interviews, the content validity of its constructs was ideal. In addition, the KMO values of each scale were greater than 0.800; the Bartlett sphericity test had a statistically significant probability (P≈0.000 < 0.001), which reached a considerable level and was suitable for factor analysis (see S2 Appendix in S1 File).

A validation factor analysis (CFA) was used to assess the quality of the measurement model. The results indicate a good fit with χ^2^ = 673.191, p ≈ 0.000, df = 413, χ^2^/df = 1.630, GFI = 0.895, CFI = 0.962, TLI = 0.958, RMSEA = 0.042 (<0.08, value is considered a good fit [85]). The standard factor loadings of all items were calculated using CFA via AMOS 24. S1 Appendix in S1 File indicates that CR values exceeded 0.8, AVE values surpassed 0.5, and standard factor loadings exceeded 0.6. The results prove that the convergent validity and internal consistency reached an acceptable level [86]. We used two methods to test the discriminant validity. First, the Fornel-Lacker criteria were used for assessment [87]. Table 1 shows that all the squared roots of the AVE are larger than the corresponding latent variables correlations. And then, we tested the discriminant validity using the Heterotrait-Monotrait Ratio of Correlations (HTMT). The results are shown in Table 2. All HTMT values are less than 0.85, indicating that good discriminant validity can be established between the variables in this study.

**Table 1:** Measure correlations, means, standard deviations (SD).

Variables	Mean	S.D.	AI	RI	MI	RDC	TCC	TIP	AC
**Affective interaction (AI)**	3.875	1.439	**0.876**						
**Resource interaction (RI)**	4.013	1.172	0.382***	**0.758**					
**Management interaction (MI)**	4.159	1.066	0.385***	0.592***	**0.709**				
**R&D capability (RDC)**	4.152	1.130	0.389***	0.610***	0.474***	**0.799**			
**TC capability (TCC)**	4.108	1.119	0.385***	0.655***	0.540***	0.542***	**0.723**		
**technological innovation performance (TIP)**	4.304	1.189	0.383***	0.675***	0.635***	0.499***	0.628***	**0.872**	
**Absorptive capacity (AC)**	4.274	1.204	0.241***	0.346***	0.365***	0.201**	0.359***	0.381***	**0.802**

Notes: ** P<0.05,

*** P<0.001; Values on the diagonal are the square-root of the average variance extracted for each construct (AVE).

**Table 2:** Discriminant validity test (HTMT value).

Variables	AI	RI	MI	RDC	TCC	TIP	AC
AI	1.000						
RI	0.396	1.000					
MI	0.381	0.613	1.000				
RDC	0.402	0.654	0.511	1.000			
TCC	0.381	0.756	0.537	0.560	1.000		
TIP	0.381	0.688	0.658	0.544	0.643	1.000	
AC	0.236	0.380	0.399	0.241	0.343	0.354	1.000

#### Multicollinearity test

We have seen from the measurement models how the constructs measures used in this study are reliable and valid. The next step is an evaluation of the structural model before moving on. It is vital to examine the level of collinearity in the structural model. SPSS 25.0 was used for collinearity assessment. Table 3 indicates no multicollinearity problems [88], as the values of tolerance are above the 0.5 thresholds, and all values of variance inflation factor (VIF) are below the threshold of 2.

**Table 3:** Assessment of multicollinearity.

Variable	TIP as Dependent variable		RDC as Dependent variable		TCC as Dependent variable	
	Tolerance	VIF	Tolerance	VIF	Tolerance	VIF
Affective interaction	0.806	1.24	0.837	1.195	0.837	1.195
Resource interaction	0.520	1.923	0.668	1.496	0.668	1.496
Management interaction	0.659	1.518	0.669	1.495	0.669	1.495
Absorptive capacity	——		0.846	1.183	0.846	1.183
R&D capability	0.610	1.640	——			
TC capability	0.832	1.583				

## Results of regression analysis

Models 1 to 3 in Table 4 depict the effects of enterprise interaction (affective interaction, resource interaction, and management interaction) on technological innovation performance, respectively. The coefficients are all greater than 0 and p = 0.000<0.001, indicating that enterprise interaction has a significant positive effect on technological innovation performance. H1a, H1b, and H1c were supported. Models 4~9 present the results of multiple regressions of the three dimensions of enterprise interaction and the two dimensions of technological innovation capability, respectively. According to the empirical results, the dimensions of enterprise interaction have a significant positive effect on both R&D capability and TC capability, and H2a, H2b, H2c, H3a, H3b, and H3c are supported.

**Table 4:** Results of enterprise interaction and technological innovation performance, technological innovation capability.

	Technology innovation performance			R&D capability			TC capability		
	Model1	Model2	Model3	Model4	Model5	Model6	Model7	Model8	Model9
**Control variables**									
Year	0.020	0.011	0.001	0.054	0.045	0.037	0.059	0.050	0.043
Size	0.035*	0.021*	0.022*	0.018*	0.006*	0.010*	0.028*	0.017*	0.019*
Sales	0.008	0.002	-0.008	-0.005	-0.011	-0.020	0.009	0.003	-0.005
**Independent variables**									
Affective interaction	0.293*** (7.096)			0.284*** (7.273)			0.260*** (6.655)		
Resource interaction		0.623*** (14.634)			0.548*** (13.006)			0.525*** (12.384)	
Management interaction			0.648*** (13.420)			0.465*** (9.203)			0.466*** (9.334)
R^2^	0.127	0.379	0.339	0.135	0.328	0.198	0.115	0.306	0.201
Adj R^2^	0.117	0.372	0.332	0.125	0.320	0.189	0.105	0.298	0.192
F	12.797***	53.835***	45.303***	13.767***	42.985***	21.758***	11.510***	38.901***	22.263***

Notes: * P<0.01,

** P<0.05,

*** P<0.001; t-statistics are in parentheses.

### Mediating effect test

As indicated above, the direct path between enterprise interaction, technological innovation capability, and technological innovation performance is significant. Therefore, the inclusion of customer satisfaction as a mediator is meaningful. And the mediation model was estimated via bootstrapping. As seen from Table 5, in the test of all mediation effects, the Bootstrap result did not contain 0 within the 95% confidence interval, indicating that all mediation paths were significant. At the same time, it is crucial to find out the strength of mediation. The strength of mediation is computed via variance accounted for (VAF), as Hadi et al. (2016) suggested [89]. Table 5 reveals that 42.61% of the effect of affective interaction on technology innovation performance is explained via R&D capability. As the value of VAF is between 20% and 80%, R&D capability partially mediates the relationship between affective interactions and technology innovation performance. As can be seen from the following table, VAF values of all intermediary paths are between 20%-80%, indicating the existence of partial intermediary utility. Therefore, R&D capability partially mediates the relationship between enterprise interactions and technology innovation performance; hypothesis 4a is partially verified. TC capability partially mediates the relationship between enterprise interactions and technology innovation performance; hypothesis 4b is partly supported.

**Table 5:** Mediation analysis by Bootstrap and result of VAF.

Mediator	Path	Direct effect	Indirect effect	Total effect	VAF	T values	LLCL	ULCL
R&D capability	AI→TIP	0.167***	——	0.291	42.61%	4.169	0.088	0.246
	AI→R&D→TIP	——	0.284×0.437 = 0.124***			5.391	0.081	0.171
	RI→TIP	0.405***	——	0.522	22.41%	10.016	0.406	0.604
	RI→R&D→TIP	——	0.549×0.216 = 0.117***			3.901	0.062	0.180
	MI→TIP	0.507***	——	0.649	21.88%	9.976	0.407	0.607
	MI→R&D→TIP	——	0.466×0.304 = 0.142***			5.071	0.091	0.202
TC capability	AI→TIP	0.156***	——	0.292	46.58%	4.134	0.082	0.230
	AI→TC→TIP	——	0.258×0.526 = 0.136***			5.231	0.086	0.190
	RI→TIP	0.448***	——	0.624	28.21%	9.351	0.354	0.542
	RI→TC→TIP	——	0.525×0.335 = 0.176***			5.333	0.114	0.240
	MI→TIP	0.463***	——	0.648	28.55%	9.445	0.367	0.560
	MI→TC→TIP	——	0.468×0.396 = 0.185***			5.442	0.125	0.257

Notes: * P<0.01,

** P<0.05,

*** P<0.001.

### Moderating effect test

**Fig 3:**
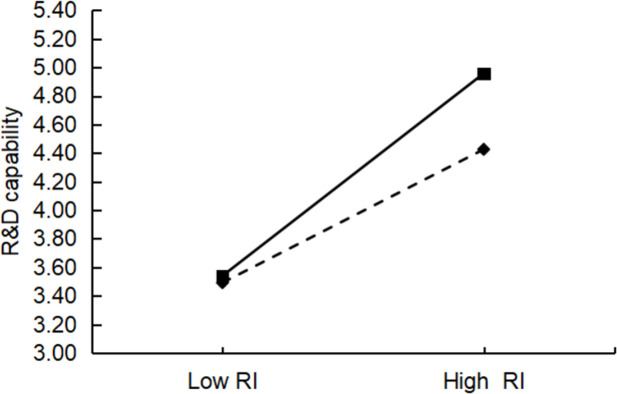
Test of the moderating effect of absorptive capacity differences on resource interaction and R&D capabilities. The dashed line represents low absorptive capacity differences while the solid line represents high absorptive capacity differences (same below).

**Fig 4:**
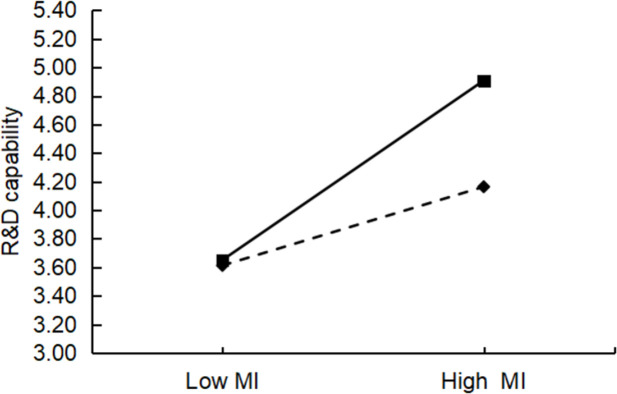
Test of the moderating effect of absorptive capacity differences on management interaction and R&D capabilities.

**Fig 5:**
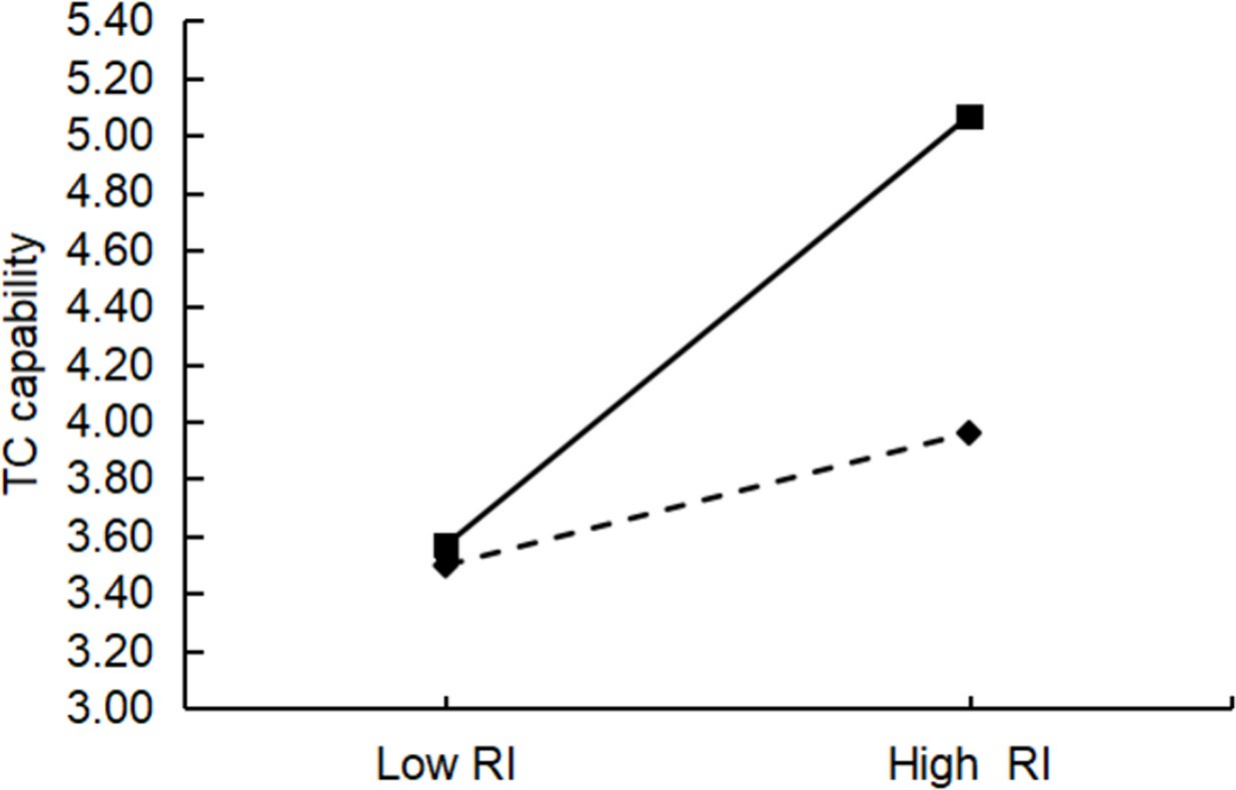
Test of the moderating effect of absorptive capacity differences on resource interaction and TC capabilities.

**Fig 6:**
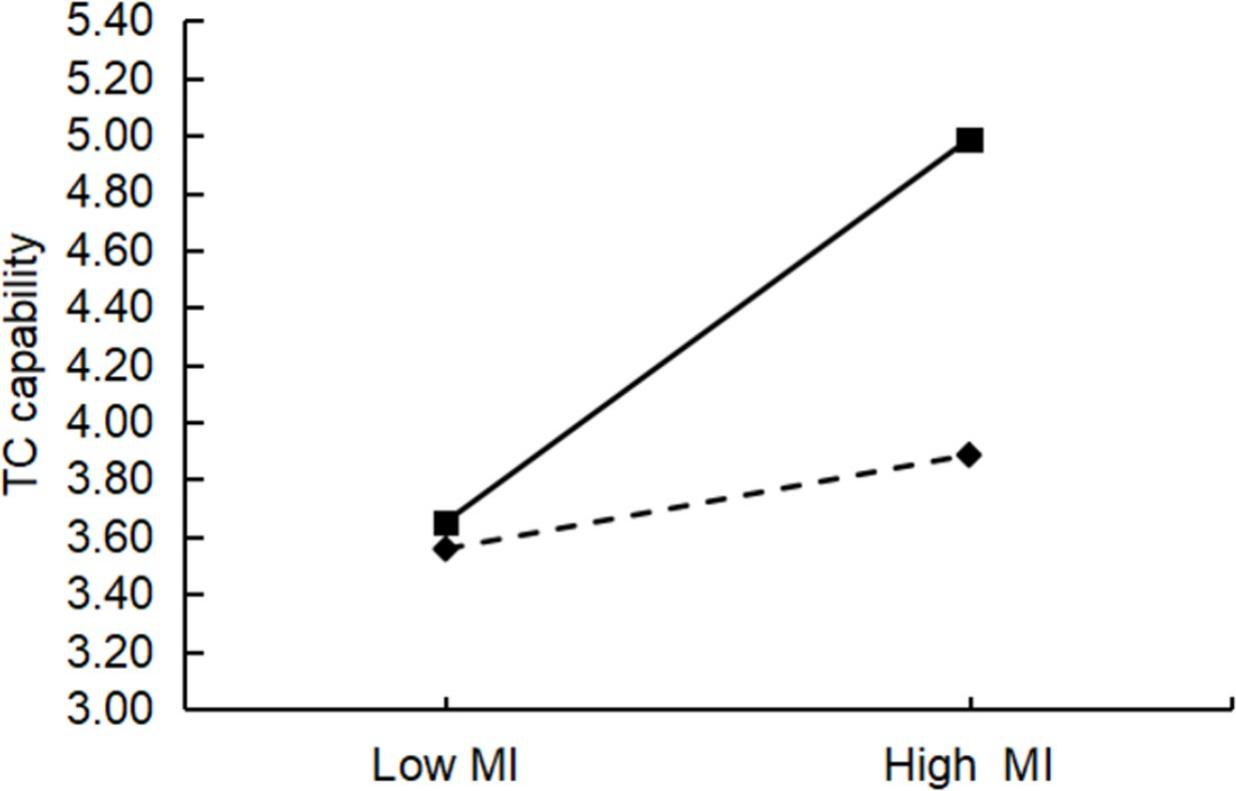
Test of the moderating effect of absorptive capacity differences on management interaction and TC capabilities.

This study used hierarchical regression to test for moderating effects, and all data were standardized to avoid multicollinearity. Table 6 shows the results of the hierarchical regression. In the table below, the coefficients of the interaction terms for Model 11 and Model 17 are not significant, indicating that there is no moderating effect of absorptive capacity between affective interaction and technological innovation capability (R&D capability, TC capability), which does not support H6a and H6b. The coefficients of the interaction terms for the rest of the models are significant, and all the AdjR^2^ values have different degrees of increase compared to the model without interaction. For example, the AdjR^2^ value also increased by 0.064 in Model 19 compared with Model 18, indicating a significant moderating effect of absorptive capacity. Therefore, H6c, H6d, H6e, and H6f were supported. Furthermore, a slope analysis was conducted using the limits of one standard deviation above and one standard deviation below the variable mean. These analyses illustrated the impact of resource interaction and management interaction on technological innovation capabilities (R&D and TC) when there are absorptive capacity differences (Figs 3–6). The figure shows that the o absorptive capacity difference had a clear moderating effect.

**Table 6:** Results of moderating effect.

	R&D capability						TC capability					
	M10	M11	M12	M13	M14	M15	M16	M17	M18	M19	M20	M21
**Control variables**												
Year	0.050	0.048	0.040	0.033	0.035	0.022	0.056	0.055	0.047	0.033	0.042	0.025
Size	0.022*	0.024*	0.007*	0.003*	0.012*	0.009*	0.035*	0.036*	0.021*	0.013*	0.025*	0.020*
Sales	-0.007	-0.005	-0.011	-0.009	-0.019	-0.015	0.004	0.005	0.001	0.004	-0.006	-0.002
**Independent variables**												
AI	0.330*** (6.535)	0.316*** (6.171)					0.284*** (5.661)	0.273*** (5.357)				
RI			0.558*** (12.032)	0.520*** (10.998)					0.509*** (10.901)	0.439*** (9.560)		
MI					0.414*** (8.137)	0.399*** (7.999)					0.389*** (7.759)	0.370*** (7.660)
**Moderator**												
AC	0.145** (2.877)	0.184** (3.247)	0.032 (0.690)	0.108* (2.092)	0.072 (1.414)	0.157** (2.919)	0.231** (4.618)	0.262** (4.646)	0.124** (2.662)	0.263*** (5.263)	0.157** (3.115)	0.269** (5.151)
AI×AC		0.071 (1.496)						0.057 (1.195)				
RI×AC				0.125** (3.214)						0.229** (6.072)		
MI×AC						0.169*** (4.130)						0.222*** (5.614)
R^2^	0.155	0.160	0.328	0.348	0.203	0.239	0.166	0.169	0.320	0.384	0.223	0.287
Adj R^2^	0.143	0.146	0.319	0.337	0.191	0.226	0.154	0.155	0.310	0.374	0.212	0.275
F	12.895***	11.157***	34.432***	31.176***	17.856***	18.401***	14.002***	11.921***	33.075***	36.515***	20.190***	23.537***

Notes: * P<0.01,

** P<0.05,

*** P<0.001; t-statistics are in parentheses.

### Robust test

This study uses a similar variable replacement method to verify the robustness of the research results [90]. Under the premise of keeping the original analysis model unchanged, dividing continuous independent variables (affective interaction, resource interaction, and management interaction) into large and small levels according to the median, and using dummy variables to replace independent variables for the regression model analysis. As seen from Table 7, the direction and significance of the critical coefficients of each variable have not changed, indicating that the robustness of the research results is promising.

**Table 7:** The results of robust test.

	Technology innovation performance		
	Model22	Model23	Model24
**Control variables**			
Year	0.020	0.023	0.003
Size	0.034*	0.028*	0.029*
Sales	0.004	0.001	-0.006
**Independent variables**			
Affective interaction (MAX)	0.347*** (6.768)		
Resource interaction (MAX)		0.732*** (10.844)	
Management interaction (MAX)			0.719*** (10.426)
R^2^	0.117	0.252	0.237
Adj R^2^	0.107	0.243	0.228
F	11.658***	49.643***	37.416***

Notes: * P<0.01,

** P<0.05,

*** P<0.001; t-statistics are in parentheses.

## Conclusion and discussion

### Research results

This paper introduces the concept of interaction from the marketing and supply chain fields to the more pervasive innovation network. It combines the ARA model to classify enterprise interaction into three dimensions: affective interaction, resource interaction, and management interaction, at three levels: perception, behavior, and maintenance. Based on the theoretical analysis, a theoretical model with enterprise interaction in innovation networks as the independent variable, technology innovation performance as the dependent variable, R&D capability, TC capability as the mediator, and absorptive capacity as the moderator was constructed. The theoretical model was empirically tested using a large sample data analysis method, and the following conclusions were drawn.

1. Three dimensions of enterprise interaction, that is, affective interaction, resource interaction, and management interaction, directly impact technological innovation performance. (2) Two dimensions of technological capability innovation (R&D capability and TC capability) mediate between enterprise interaction and technological innovation performance. This finding further confirms the logic of the resource-based theory of "resource→capability→performance." (3) The moderating effect of absorptive capacity between resource interaction, management interaction, and technological innovation capability is significant. However, the moderating impact between affective interaction and technological innovation capability is not statistically significant. One possible reason is that affective interaction, as a foundation for firms to build a good relationship network, is more helpful in communicating to understand their own needs and those of other firms and has yet to result in substantial resource support. Therefore, it isn’t easy to obtain valuable technical knowledge. Hence, the role of absorptive capacity in converting "affective resources" into "capabilities" is not particularly obvious. On the contrary, resource interaction and management interaction broaden the quantity and quality of external information resources through resource sharing, joint problem-solving, etc. Enterprises with high absorptive capacity tend to have higher information search and processing ability, facilitating the transfer of invisible knowledge and thus better enhancing their technological development and product commercialization skills.

### Contributions

#### Theoretical contribution

1. This study reveals the intrinsic mechanism by which enterprise interaction in innovation networks affect technological innovation performance, thus deepening the theoretical study of the intrinsic link between network relationships and technological innovation performance. Combined with existing studies, the academic circle has affirmed the research on the network and inter-organizational levels. However, there needs to be more research on the individual enterprise level. In particular, more theoretical and empirical research needs to be conducted on how the interaction between enterprises affects technological innovation performance. The study discusses the direct influence of enterprise interaction on firm’s technological innovation performance and reveals the mediating role of technological innovation capability between enterprise interaction and technological innovation performance. The moderating effect of absorptive capacity on enterprise interaction and technological innovation capability is also verified. In a certain sense, it enriches the research on the theory of a firm’s network relations technological innovation.
2. This study expands the theoretical research of enterprise interaction to a certain extent. Currently, the research on interaction is carried out from the perspective of the dual relationship between buyer-suppliers and customers [25, 26]. The research scope also focuses on the cooperation relationship, cooperation performance, customer performance, and other aspects. There are few pieces of literature on the impact of interaction on actors at the micro level. The study defines the connotation of enterprise interactions based on resource-related theory, combines the characteristics of innovation networks, divides the dimensions of enterprise interactions, and forms a measurement scale. Focusing on the actor of interaction, the study uses empirical methods to reveal the influence mechanism of interaction on their innovation activities. This research enriches and expands the theoretical analysis of enterprise interaction in a certain sense.

#### Practical implications

This study provides a new management perspective on the practice of technology innovation management in enterprises. Network relationships are a significant feature of today’s business environment. In innovation networks, innovation is no longer an independent activity that occurs within a firm’s organization; the innovating firm needs to pay more attention to the resources and capabilities of other firms in the network [16] and how to combine its resources with those of other firms effectively. Strengthening the positive influence of enterprise interaction on technological innovation ability by improving absorptive capacity. The active interaction and cooperation of members in an innovation network are critical to the success or failure of technological innovation.

The study focuses on the impact of the interaction behavior of member firms in innovation networks on innovation performance. Studying enterprise interaction’s connotation and structural dimensions can help firms understand the behavioral logic behind the interactions. By revealing the impact of enterprise interactions on their technological innovation, it can help firms improve their perceptions of the importance of network relationships and interaction behaviors. Research on the moderating effect of absorptive capacity is helpful in promoting the transformation of enterprise interaction to technological innovation capability. In addition, the research allows firms to wedge into innovation networks better and maintain good interaction with network members in their technological innovation practices. It leads to better access to external innovation resources and improves firms’ technological innovation performance.

#### Administration recommendations

1. Give full play to the role of enterprise interaction in promoting technological innovation activities. This study shows that enterprise interaction positively improves technical innovation ability and technological innovation performance [53, 91]. Therefore, in practice, enterprises should pay attention to improving the skills of affective interaction, resource interaction, and management interaction. In terms of affective interaction, companies should take the initiative to communicate with other companies in the innovation network formally or informally. Creating a good interaction atmosphere helps reduce each other’s defensiveness and stimulates partner firms’ enthusiasm for interaction and willingness to cooperate. Resource interaction is the substantive behavior carried out in the interaction process. Enterprises should make the sharing and exchange process of resources transparent to avoid the "trust breach" caused by information asymmetry and other reasons. In addition, enterprises should promote the integration of new resources obtained through interaction with internal resources and optimize the existing resource mix to meet the needs of different types of innovation and development. For management interaction, enterprises need to dynamically adjust and optimize the way and frequency of mutual interaction according to the needs of cooperation and actively guide the establishment of standard behavior norms to reduce opportunism. Develop conflict resolution mechanisms to improve the speed and efficiency of conflict resolution, mitigate related risks and uncertainties, and reduce transaction costs. Ultimately, promote high-technology innovation capabilities and performance [44].
2. Build industrial chains and ecosystems in conjunction with corporate realities and technological goals. In practice, enterprises continue to establish diverse and extensive external connections to get innovation resources while struggling to make good use of these complex external connections to avoid systemic risks. When leveraging interactions to obtain resources, companies need to pay attention to the current state of their innovation capabilities and evaluate external partners. Lay out the industry chain and ecosystem scientifically and rationally. Companies need to strengthen their understanding through interactions, focus their limited time and energy on quality resources, maintain a moderate network of relationships [92], play "relationship advantages," and avoid "relationship traps."
3. In conjunction with the research in this study, the development of absorptive capacity has a moderating role between enterprise interaction and technological innovation capabilities. Therefore enterprises should pay full attention to the value of absorptive capacity. For example, establish an internal knowledge management system and hold intra-enterprise knowledge and information sharing activities (thematic knowledge competitions, brainstorming, etc.). Form a good learning and cooperation atmosphere within the enterprise. By cultivating employees’ knowledge acquisition and application ability, the absorption capacity of the enterprise can be improved [67]. To further enhance the role of interaction in promoting technological innovation capabilities.

### Research limitations and prospects

In terms of research design, this study mainly examines the high-technology industry and the traditional manufacturing industry. Although these industries have great potential for technological innovation, this study excludes industry segmentation. Hence the findings should be applied with caution to other settings. Therefore, specific industries can be discussed in future studies to address the differences in the interactive behavior of different industries.

In terms of research content, the role of enterprise interaction on technological innovation capabilities is also influenced by factors such as relational intimacy and the quality of network relationships. This study considers enterprise interaction as an important source of external innovation knowledge and is limited by the space and research model to consider only the effect of absorptive capacity. Future research can consider verifying these factors’ effects on this study’s model to make the model richer.

Regarding model results, this study focuses on the impact of enterprise interactions on their technological innovation performance. However, interaction is a mutual process involving multiple individuals. Future research could consider extending the outcome variables to relational performance to measure the impact of interaction frequency, interaction quality, and interaction mode on inter-firm collaborative innovation performance.

## Supporting information

S1 File (RAR)
